# Reconstructive outcome analysis of the impact of neoadjuvant chemotherapy on immediate breast reconstruction: a retrospective cross-sectional study

**DOI:** 10.1186/s12885-021-08256-y

**Published:** 2021-05-08

**Authors:** Jia-Ruei Yang, Wen-Ling Kuo, Chi-Chang Yu, Shin-Cheh Chen, Jung-Ju Huang

**Affiliations:** 1 Department of Plastic and Reconstructive Surgery, Chang Gung Memorial Hospital, Linkou Medical Center, 5, Fu-Hsing Street, Kweishan, Taoyuan, 33305 Taiwan; 2 Department of General Surgery, Chang Gung Memorial Hospital, Linkou Medical Center, Taoyuan, Taiwan; 3 College of Medicine, Chang Gung University, Taoyuan, Taiwan; 4 Center for Tissue Engineering, Chang Gung Memorial Hospital, Linkou Medical Center, Taoyuan, Taiwan

**Keywords:** Breast cancer, Immediate breast reconstruction, Mastectomy, Neoadjuvant chemotherapy, Surgical complication, Aesthetic outcome

## Abstract

**Background:**

Neoadjuvant chemotherapy (NACT) was initially applied to locally advanced breast cancer to convert advanced lesions to an operable status. Currently, its application has been expanded to enhance overall oncological results, especially in patients with triple-negative or HER-2-positive breast cancer. With more NACT being applied, the role and impact of this approach on breast reconstruction needs to be determined. This study aimed to perform a complete reconstructive outcome analysis of patients receiving NACT who underwent immediate breast reconstruction.

**Methods:**

A retrospective review of a single reconstructive surgeon’s immediate breast reconstructions performed from July 2008 to December 2018 was undertaken. The results were stratified by the use of NACT. Patient demographics, delivery of NACT, adjuvant treatment, incidence of surgical complications, and postoperative photographs were analyzed.

**Results:**

A total of 269 patients were included. The mean follow-up was 46.3 months. Forty-six out of 269 patients received NACT and were included in the NACT group. The other patients were included in the non-NACT group. When implant-based reconstruction was planned, the NACT group had a higher rate of two-stage tissue expander-implant reconstruction than direct-to-implant reconstruction (*p* < 0.001). The requirement for postmastectomy radiotherapy was higher in the NACT group (*p* < 0.001). The surgical complication rates were similar between groups after adjusting for confounding factors. The objective aesthetic outcomes assessed by 6 plastic surgeons were also similar between groups.

**Conclusions:**

Immediate breast reconstruction is a safe and reliable procedure, with an acceptable reconstructive complication rate and satisfactory aesthetic outcomes, for patients treated with NACT.

**Supplementary Information:**

The online version contains supplementary material available at 10.1186/s12885-021-08256-y.

## Background

Neoadjuvant chemotherapy (NACT) is increasingly provided since 1970. NACT initially served three goals. First, it provides effective systemic treatment that is equivalent to adjuvant therapy to prevent cancer recurrence [1]. Second, neoadjuvant chemotherapy is effective at downstaging tumor volume and allows de-escalating surgery for larger tumors and/or axillary nodal involvement [1]. NACT reduces the need for axillary lymph node dissection (ALND) among patients presenting with nodal metastases at diagnosis [2], facilitates less extensive surgeries, such as breast conservation surgery (BCS), and minimizes postoperative complications, such as lymphedema. Furthermore, NACT allows for the assessment of chemosensitivity at an earlier stage in the treatment process [3].

Despite its initial role in managing locally advanced breast cancer, NACT is now widely provided for patients with triple-negative breast cancer (TNBC), human epidermal growth factor receptor-2-positive (HER-2 positive) breast cancer, and early stage breast cancer [3–6]. In 2012, an International Consensus Conference documented that neoadjuvant chemotherapy could generally be considered for every patient who is a candidate for adjuvant chemotherapy [7]. Moreover, the Gallen International Breast Cancer Conference in 2017 defined neoadjuvant therapy as the preferred treatment approach for stage II/III, triple-negative, and HER2-positive breast cancer [1].

With the increased delivery of NACT, surgical management can become more versatile. NACT helps reducing the volume of the primary tumor and the regional nodes, thus making more options for mastectomy possible, such as BCS. This allowed up to one-third of patients to be eligible for BCS, for whom mastectomy was initially indicated [6, 8]. Nevertheless, total mastectomy is still required for multifocal or extensive breast cancer patients. Taking oncologic safety into consideration, skin-sparing mastectomy (SSM) or even nipple-sparing mastectomy (NSM) can be safely performed for patients following neoadjuvant treatment [9, 10].

With more NACT regimens being applied, the impact of this approach on immediate breast reconstruction needs to be determined. Regarding surgical complications, chemotherapeutic agents may prolong wound healing with a delayed inflammatory phase of healing, reduced fibrin deposition and collagen synthesis, and delayed wound contraction [11]. Chemotherapy also destroys the patients’ immune system and increases the risk of wound infections [12]. Thus, concerns exist regarding an increase in postoperative complications after NACT, such as delayed wound healing or increased susceptibility to infections. As immediate breast reconstruction is now the standard of care in early-stage breast cancer surgeries, the impact of NACT on the outcomes of immediate breast reconstruction needs to be further defined. This issue remains controversial, and highly variable results have been reported [13–16]. In addition, the aesthetic outcome is equally important, as complications may negatively affect the aesthetic outcome. Compared with patient-reported aesthetic outcomes, objective analyses of aesthetic outcomes remain insufficient for patients who receive neoadjuvant chemotherapy and immediately undergo breast reconstruction. We therefore aimed to perform a complete reconstructive outcome analysis for both reconstructive complications and objective aesthetic outcomes in patients who received NACT and immediately underwent breast reconstruction. Of note, since the development of NACT has broadened its application in different indications inclusion patients with small tumor with several specific types, poor response to the NACT may still happen in patients receiving NACT requiring more extensive mastectomy and postoperative management. Therefore, our comprehensive review of all the patients during a long time period will provide information in patients both with good response to NACT and in more advanced stage who were not well responsive to NACT.

## Methods

### Study sample

A total of 269 patients undergoing SSM or NSM received immediate breast reconstruction performed by a single surgeon (J.H.) at Chang Gung Memorial Hospital from July 2008 to June 2018. Forty-six of 269 patients received NACT. Patients with a history of any breast surgery, previous partial mastectomy, inflammatory or recurrent breast cancer and a preoperative diagnosis of metastatic disease were excluded.

### Study design

After the study was approved by the institutional review board (202000257B0), data were retrospectively collected from electronic medical records. The patients were divided into two groups: Group 1 (NACT group) received neoadjuvant chemotherapy and mastectomy followed by immediate reconstruction, and Group 2 (non-NACT control group) underwent mastectomy and immediate reconstruction without neoadjuvant chemotherapy. Patient demographics, surgical characteristics, and reconstructive outcomes, including surgical complications and aesthetic outcomes, were collected and analyzed.

The patient demographics included age at surgery, body mass index (BMI), comorbidities (i.e., diabetes mellitus and hypertension), and history of smoking. In addition, the clinical information included pathological stage, histology, biological type with estrogen and progesterone receptor status, TNBC, HER-2 positive status, duration of follow-up after reconstruction, and adjuvant therapy including radiotherapy, chemotherapy and hormone therapy. Pathological complete response (pCR) was defined as no residual invasive breast cancer in the histopathological specimen of the breast and axillary lymph nodes (ypT0/ypTisN0).

The surgical characteristics mainly included type of mastectomy, details of sentinel lymph node (SLN) biopsy and ALND, types of immediate reconstruction, either implant-based reconstruction or autologous tissue transfer, and length of hospital stay. Details regarding the specimen size, flap size, and implant size were also recorded. Although the reconstruction method was decided by the patients and surgeon together, when patients with planned or anticipated to receive radiotherapy, implant-based reconstruction were planned as a two-stage reconstruction with tissue expander insertion first.

### Surgical complications

Surgical complications were defined as complications that were related to surgeries, including reconstruction, and were categorized as short-term (within 30 days after surgery) and late (presented 30 days after surgery) complications. Complications included infection that required antibiotic treatment, poor wound healing that required surgical intervention and wound repair, mastectomy skin flap or nipple necrosis requiring surgical debridement, hematoma or seroma required drainage, expander or implant exposure managed surgically, reconstructive flap re-exploration and implant failure. Late Complications also included capsular contracture, and implant rupture or failure.

### Aesthetic outcome

Aesthetic outcomes were evaluated using a single-blind study-specific questionnaire which blinded raters to the patients’ demographic information and surgical characteristics. To reflect the patient’s real concern for the appearance of breasts, the more precise and individualized description is required. According to the author’s clinical experience, five important questions were comprised in this questionnaire, including the shape of the reconstructed breast, symmetry of the bilateral infra-mammary fold (IMF), symmetrical bilateral breast volume and bilateral breast shape, and overall reconstruction outcomes, including skin quality, and the rates were judged by the patients’ subjective aesthetic feelings. An objective assessment of the aesthetic outcome was performed by six board-certified plastic surgeons. None of the 6 plastic surgeons involved in any of the patients’ care. Follow-up photos taken at least 6 months after surgery (or 6 months after radiotherapy if PMRT was delivered) were available in 187 of the 296 patients and were used for the evaluation. After concealing the patients’ demographic information and surgical characteristics, six qualified plastic surgeons screened 187 patients’ postoperative photographs after at least 6 months of follow-up and scored them as 1 (very dissatisfied), 2 (dissatisfied), 3 (neutral), 4 (satisfied) or 5 (very satisfied) according to the questions of the questionnaire.

### Statistical analysis

Categorical variables were compared using chi-square or Fisher’s exact tests. Continuous variables were assessed using Student’s *t* test. To analyze associations between NACT and surgical complications, multivariable logistic regression analysis was performed. According to previous literature [17–20], known confounding factors affecting surgical complications, including age, BMI, diabetes mellitus, history of smoking, and postmastectomy radiotherapy (PMRT), were analyzed. In addition, based on clinical experience, T and N stages were also investigated. This analysis allowed for a more accurate comparison of reconstructive complication outcomes. Adjusted odds ratios (ORs) and 95% confidence intervals (CIs) were calculated for NACT. Statistical analyses were performed using SPSS 24 software (IBM, Chicago, IL, USA), with *p* values less than 0.05 indicating significance.

## Results

### Patient demographics

From July 2008 to June 2018, a total of 269 patients undergoing SSM or NSM with immediate breast reconstruction by a single surgeon (J.H.) were included. Forty-six patients received NACT, whereas 223 patients who underwent immediate breast reconstruction without NACT served as the control group. The patient demographic characteristics are shown in Table 1.

**Table 1:** Summary of demographics of patients with and without NACT

Variable	All (***n*** = 269)	NACT (%) (***n*** = 46)	Non-NACT (%) (***n*** = 223)	***p*** value
Mean age at diagnosis ± SD, year	44.3 ± 8.2	40.9 ± 7.4	45.0 ± 8.2	0.002*
Mean BMI ± SD, kg/m^2^	22.9 ± 3.7	22.8 ± 3.2	22.9 ± 3.8	0.892
History of smoking	5 (1.9)	1 (2.2)	4 (1.8)	1.000
Hypertension	13 (4.8)	0 (0.0)	13 (5.8)	0.134
Diabetes mellitus	3 (1.1)	1 (2.2)	2 (0.9)	0.432
Stage				
T stage				1.000
T0-T1	95 (35.3)	30 (65.2)	144 (64.6)	
T2-T4	174 (64.7)	16 (34.8)	79 (35.4)	
N stage				0.042*
N0^†^	187 (69.5)	25 (54.3)	162 (72.6)	
N1^†^	62 (23.0)	16 (34.8)	46 (20.6)	
N2-N3	20 (7.4)	5 (10.9)	15 (6.7)	
Pathological stage				< 0.001*
0^†^	52 (19.9)	0 (0.0)	52 (23.3)	
I	85 (32.6)	12 (31.6)	73 (32.7)	
II	98 (37.5)	19 (50.0)	79 (35.4)	
III	26 (10.0)	7 (18.4)	19 (8.5)	
Histological type				
IDC^†^	167 (62.1)	39 (84.8)	128 (57.4)	< 0.001*
ILC	21 (7.8)	1 (2.2)	20 (9.0)	0.141
DCIS^†^	109 (40.5)	0 (0.0)	109 (48.9)	< 0.001*
LCIS	3 (1.1)	0 (0.0)	3 (1.3)	1.000
Other	32 (11.9)	4 (8.7)	28 (12.6)	0.619
pCR		8 (17.4)		
Estrogen receptor positive	217 (81.0)	37 (80.4)	180 (81.1)	1.000
Progesterone receptor positive	206 (76.9)	34 (73.9)	172 (77.5)	0.571
TNBC/HER-2 positive	86 (32.0)	23 (50.0)	63 (28.3)	0.005*
HER-2 amplified	73 (27.3)	20 (43.5)	53 (24.0)	0.010*
Triple negative breast cancer	13 (4.8)	3 (6.5)	10 (4.5)	0.471
Post-mastectomy radiotherapy	52 (19.3)	30 (65.2)	22 (9.9)	< 0.001*
Adjuvant chemotherapy	150 (59.1)	32 (69.6)	127 (57.0)	0.139
Adjuvant hormone therapy	203 (75.5)	35 (76.1)	168 (75.3)	1.000
Local recurrence	1 (0.4)	0 (0.0)	1 (0.4)	1.000
Distant metastasis	16 (5.9)	9 (19.6)	7 (3.1)	< 0.001*
Interval between NACT and surgery, week		4.8 ± 2.6		
Mean follow-up ± SD, month	46.3 ± 32.2	35.0 ± 28.5	48.6 ± 32.5	0.009*

*NACT* neoadjuvant chemotherapy, *SD* standard deviation, *BMI* body mass index, *pCR* pathological complete response

^†^The adjusted standardized residual was greater than 2 which indicates the column proportions were significantly different at *p* < 0.05 level

Patients in the NACT group were significantly younger than those in the non-NACT group (40.9 ± 7.4 vs. 45.0 ± 8.2 years old; *p* = 0.002), with more patients in the NACT group had an advanced cancer stage. Regarding N stage, the NACT group had a significantly higher proportion of N1 disease (34.8%) than the non-NACT group (20.6%), while the non-NACT group had a significantly higher proportion of N0 disease (72.6% versus 54.3%, *p* = 0.042). Similarly, the non-NACT group presented with a higher proportion in stage 0 disease (*p* < 0.001). Regarding the subtypes of breast cancer, the NACT group had a higher proportion of invasive ductal carcinoma (IDC) (84.8%) than the non-NACT group (57.4%) (*p* < 0.001), while the non-NACT group had a higher proportion of ductal carcinoma in situ (DCIS) (48.9%) (*p* < 0.001). Following NACT, pCR was noted in 8 (17.4%) patients. Either TNBC or HER-2 positivity was reported in 86 breast cancer patients (32%), and these classifications were significantly more common in the NACT group (50%) (*p* = 0.005).

PMRT was administered to 52 (19.3%) patients (Table 1). A significantly higher proportion of patients in the NACT group (65.2%) received PMRT than in the non-NACT group (9.9%) (*p* < 0.001). The local recurrence rate was similar between the NACT group (0.0%) and the non-NACT group (0.4%) (*p* = 1.000). A higher rate of distant metastasis was observed in the NACT group (19.6%) than in the non-NACT group (3.1%) (*p* < 0.001).

### Surgical characteristics

The surgical characteristics are shown in Table 2. The NACT group was significantly less likely to undergo SLN biopsy (*p* < 0.001); instead, more ALND were directly performed (*p* < 0.001). A tendency towards more NSM than SSM was performed in the NACT group (41.3%) in comparison to the non-NACT group (30.9%), but the difference was not statistically significant (*p* = 0.226).

**Table 2:** Summary of surgical characteristics of patients with and without NACT

Variable	All (***n*** = 269)	NACT (%) (***n*** = 46)	Non-NACT (%) (***n*** = 223)	***p*** value
Laterality, no. of patient				0.365
Unilateral	260 (96.7)	46 (100.0)	214 (96.0)	
Bilateral	9 (3.3)	0 (0.0)	9 (4.0)	
Location, no. of breast				0.872
Right	146 (52.5)	25 (54.3)	121 (52.2)	
Left	132 (47.5)	21 (45.7)	111 (47.8)	
Type of mastectomy				0.226
Skin-sparing	181 (67.3)	27 (58.7)	154 (69.1)	
Nipple-sparing	88 (32.7)	19 (41.3)	69 (30.9)	
Sentinel lymph node biopsy	230 (85.5)	26 (56.5)	204 (91.5)	< 0.001*
Positive SLNB	47 (17.5)	7 (15.2)	40 (17.9)	0.832
Axillary lymph node dissection	92 (34.2)	33 (71.7)	59 (26.5)	< 0.001*
Breast margin positive	3 (1.1)	2 (4.3)	1 (0.4)	0.077
Nipple core positive	3 (1.1)	2 (4.3)	1 (0.4)	0.077
Type of Immediate reconstruction				0.408
Implant-based reconstruction	163 (60.6)	25 (54.3)	138 (61.9)	
Autologous reconstruction	106 (39.4)	21 (45.7)	85 (38.1)	
Type of implant-based reconstruction				0.001*
Direct to implant	130 (79.8)	13 (52.0)	117 (84.8)	
Tissue expander and implant	33 (20.2)	12 (48.0)	21 (15.2)	
Type of autologous reconstruction				0.869
Muscle-sparing free TRAM	5 (4.7)	0 (0.0)	5 (5.9)	
DIEP	90 (84.9)	19 (90.5)	71 (83.5)	
PAP	11 (10.4)	2 (9.5)	9 (10.6)	
Specimen size (gm)	380 ± 200	422 ± 216	372 ± 196	0.133
Flap size (gm)	441 ± 186	387 ± 147	454 ± 192	0.136
Implant size (ml)	300 ± 90	292 ± 77	302 ± 92	0.691
Hospital stay ± SD, day	9.8 ± 8.1	9.8 ± 4.1	9.8 ± 8.8	0.990
Aesthetic revision surgery	57 (21.2)	7 (15.2)	50 (22.4)	0.326

*NACT* neoadjuvant chemotherapy, *SLNB* sentinel lymph node biopsy, *TRAM* transverse rectus myocutaneous, *DIEP* deep inferior epigastric perforator, *PAP* profunda artery perforator

**Fig. 1:**
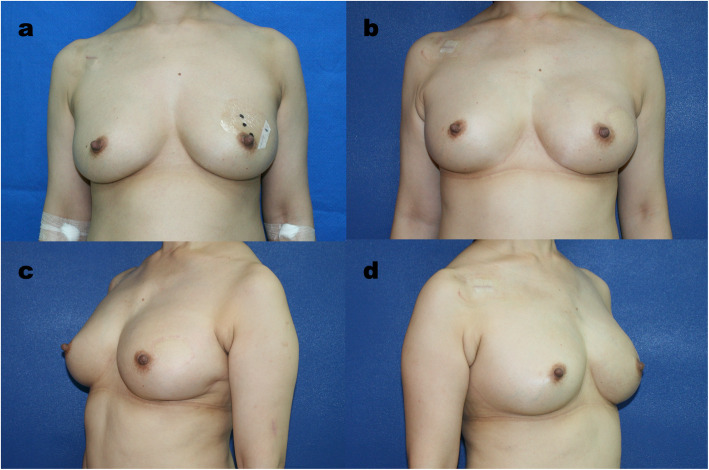
Nipple-sparing Mastectomy and Immediate Breast using Free DIEP Flap after Neoadjuvant Chemotherapy. **a** This young woman at her forties had left invasive ductal carcinoma with lymph node involvement. The clinical stage was T2N1M0. She received neoadjuvant chemotherapy with 4 cycles of Perjeta, Taxotere and Herceptin and 4 cycles of CEF. The patient’s cancer status eventually reached a clinical complete response. **b**, **c**, **d** The patient underwent left nipple-sparing mastectomy with immediate DIEP free flap reconstruction. The pathology report showed a pathological complete response. At the 14-month follow-up, the patient was satisfied with the result

**Fig. 2:**
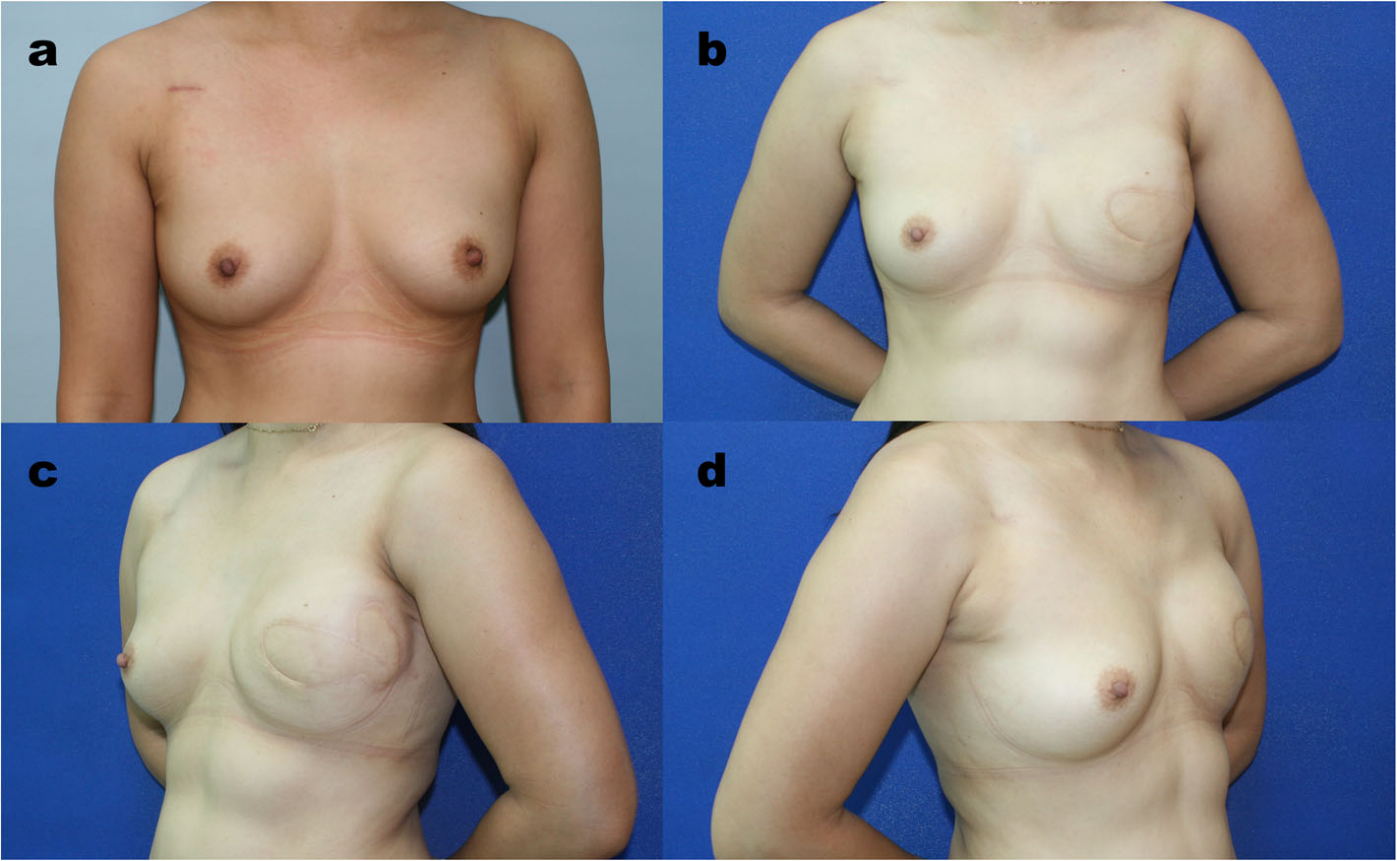
Result of Neoadjuvant Chemotherapy and Immediate Breast Reconstruction following with PMRT in Advanced Breast Cancer. **a** This is a case of left invasive ductal carcinoma with lymph node involvement. The clinical stage was T3N1M0. She received neoadjuvant chemotherapy with 4 cycles of Taxotere and epirubicin. **b**, **c**, **d** The patient initially underwent left nipple-sparing mastectomy. However, nipple core frozen section revealed cancer involvement, and the mastectomy had to be converted to skin-sparing mastectomy. As a result, a larger skin paddle was required from the DIEP flap to compensate for the mastectomy skin defect. The reconstruction was finished with a free DIEP flap immediately after mastectomy. After surgery, the patient received adjuvant chemotherapy with 3 cycles of CEF and 3 cycles of Taxotere, adjuvant radiotherapy and hormone therapy with tamoxifen. At the 39-month follow-up, an acceptable reconstructive outcome with minimal contracture and skin reactions was achieved after radiotherapy

**Fig. 3:**
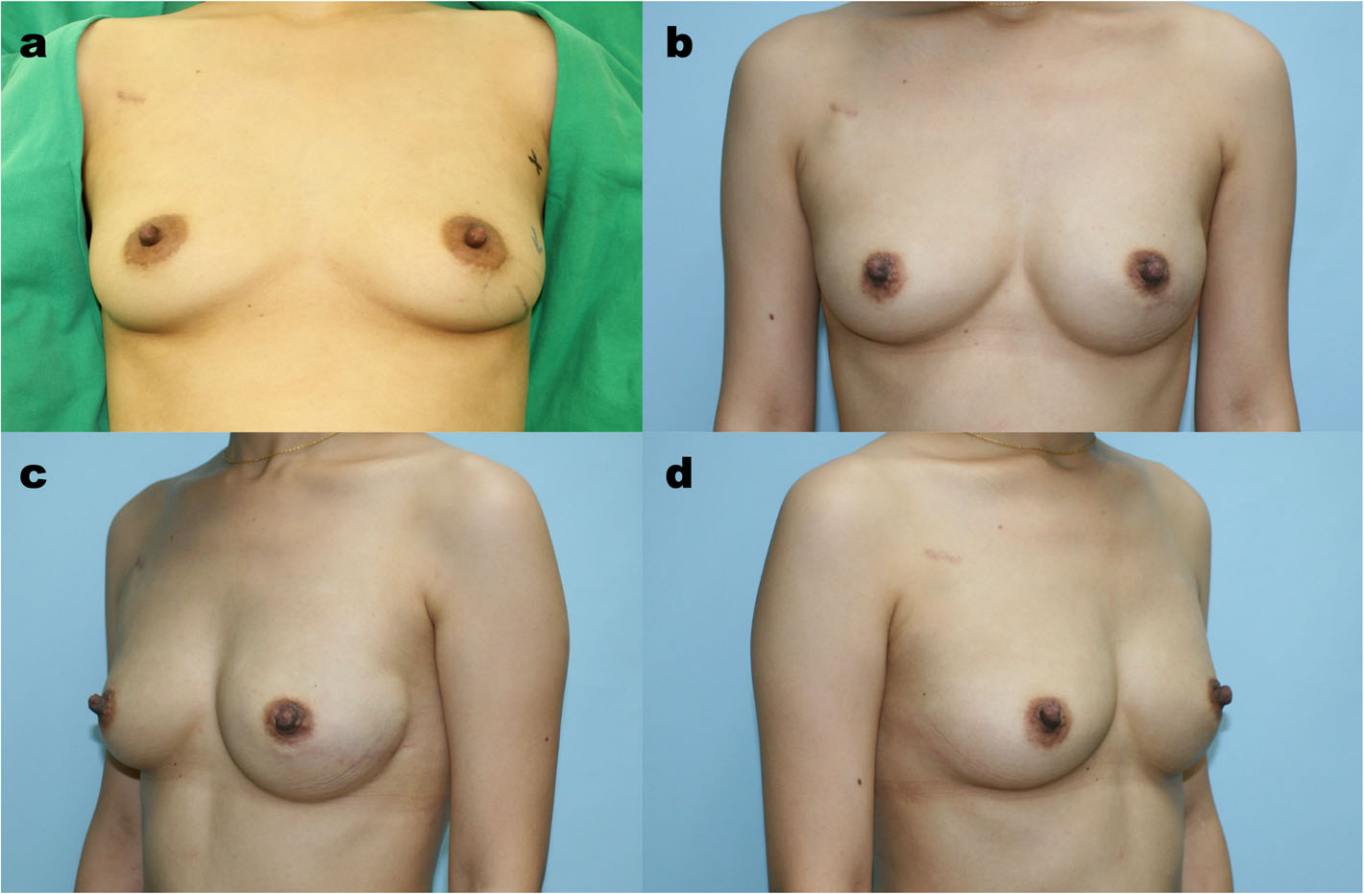
Direct-to-Implant Immediate Breast Reconstruction following with Nipple-sparing Mastectomy after Neoadjuvant Chemotherapy. **a** This 36-year-old woman had left invasive ductal carcinoma with lymph node involvement. The clinical stage was T2N1M0. She received neoadjuvant chemotherapy with 4 cycles of Taxotere, cisplatin and Herceptin and 4 cycles of CEF. **b**, **c**, **d** The patient underwent left nipple-sparing mastectomy with immediate direct-to-implant reconstruction using silicone implants. The pathology report showed a pathologic complete response. The direct-to-implant result was satisfactory at the 19-month follow-up

There was a tendency that more autologous breast reconstructions to be performed in the NACT group (45.7%) than in the non-NACT group (38.1%), but the difference was not significant (*p* = 0.408). The DIEP flap remained our first choice in both the NACT group (90.5%) and the non-NACT group (83.5%), and the PAP flap served as an alternative. Regarding implant-based reconstruction, while the direct-to-implant (DTI) approach remained our first priority, the use of a two-stage reconstruction was significantly higher in the NACT group than in the non-NACT group (48% vs. 15.2%, *p* = 0.001) (Figs. 1, 2 and 3).

### Surgical complications

Table 3 presents the results of the multivariable logistic regression analysis to determine the impact of NACT on immediate breast reconstruction. There was no significant difference in surgical complications between the NACT group and the non-NACT group, in terms of both short-term and late complications. In general, in both the NACT and non-NACT groups, the overall short-term and late surgical complication rates were between 17.3–21.7%, with wound healing problems and mastectomy skin flap partial thickness as the major short-term complications.

**Table 3:** Reconstructive complication analysis among patients with and without NACT

Short-term complication (< 30 days)	All	NACT	Non-NACT	OR (95% CI) of NACT	***p*** value
Any complication	50 (19.2)	10 (21.7)	40 (18.7)	1.56 (0.63–3.86)	0.334
Re-exploration	3 (1.2)	1 (2.2)	2 (0.9)	0.79 (0.00–145.23)	0.930
Infection	2 (0.8)	0 (0.0)	2 (0.9)	N/A	N/A
Mastectomy skin flap necrosis	15 (5.8)	3 (6.5)	12 (5.6)	1.28 (0.29–5.60)	0.743
Nipple necrosis	6 (2.3)	2 (4.3)	4 (1.9)	1.57 (0.19–13.09)	0.675
Seroma	7 (2.7)	1 (2.2)	6 (2.8)	1.58 (0.15–16.81)	0.707
Hematoma	4 (1.5)	2 (4.3)	2 (0.9)	N/A	N/A
Expander/Implant exposure	1 (0.4)	0 (0.0)	1 (0.5)	N/A	N/A
Poor wound healing	33 (12.7)	5 (10.9)	28 (13.1)	1.00 (0.32–3.10)	0.996
Implant failure	1 (0.4)	0 (0.0)	1 (0.5)	N/A	N/A
**Long-term complication (> 30 days)**					
Any complication	45 (17.3)	8 (17.4)	37 (17.3)	0.71 (0.25–1.96)	0.502
Infection	11 (4.2)	4 (8.7)	7 (3.3)	3.26 (0.69–15.26)	0.134
Expander/Implant exposure	4 (1.5)	0 (0.0)	4 (1.9)	N/A	N/A
Implant rupture	9 (3.5)	0 (0.0)	9 (4.2)	N/A	N/A
Capsular contracture	24 (9.2)	3 (6.5)	21 (9.8)	0.37 (0.08–1.67)	0.195
Contracture release	13 (5.0)	1 (2.2)	12 (5.6)	0.15 (0.01–1.93)	0.144
Implant failure	15 (5.8)	0 (0.0)	15 (7.0)	N/A	N/A

*NACT* neoadjuvant chemotherapy, *N/A* not applicable

### Aesthetic outcomes

In summary, the aesthetic outcomes were similar between two groups, except the score for overall outcome was lower in the NACT group. However, when the aesthetic outcomes were evaluated separately according to different reconstruction methods, the difference only presented in the implant-based reconstruction group. Implant-based reconstruction tended to have lower aesthetic outcome scores than autologous reconstruction in both the NACT group and the non-NACT group (Table 4). For implant-based reconstruction, compared to those in the non-NACT group, the scores for aesthetic outcomes in the NACT group were significantly lower in terms of overall outcome (3.1 ± 0.8 vs. 3.6 ± 0.7, *p* = 0.035) (Table 4). To clarify the factors affecting aesthetic outcomes, further subgroup analysis based on PMRT was conducted.

**Table 4:** Subgroup analysis of aesthetic outcome among patients with and without NACT

Outcome	All (***n*** = 187)			Implant-based reconstruction (***n*** = 86)			Autologous reconstruction (***n*** = 101)		
	NACT (***n*** = 35)	Non-NACT (***n*** = 152)	***p*** value	NACT (***n*** = 15)	Non-NACT (***n*** = 71)	***p*** value	NACT (***n*** = 20)	Non-NACT (***n*** = 81)	***p*** value
Breast shape of reconstructive site	3.6 ± 0.8	3.8 ± 0.7	0.136	3.1 ± 0.8	3.5 ± 0.8	0.092	4.0 ± 0.6	4.1 ± 0.5	0.429
Symmetry of infra-mammary fold	3.9 ± 0.7	4.0 ± 0.7	0.524	3.4 ± 0.7	3.7 ± 0.8	0.123	4.2 ± 0.5	4.1 ± 0.6	0.552
Symmetry of breast volume	3.7 ± 0.7	3.8 ± 0.7	0.575	3.4 ± 0.8	3.7 ± 0.8	0.218	4.0 ± 0.5	3.9 ± 0.6	0.628
Symmetry of breast shape	3.4 ± 0.8	3.7 ± 0.8	0.128	3.0 ± 0.8	3.5 ± 0.9	0.072	3.8 ± 0.6	3.8 ± 0.6	0.622
Overall outcomes	3.6 ± 0.8	3.8 ± 0.7	0.052	3.1 ± 0.8	3.6 ± 0.7	0.035*	3.9 ± 0.6	4.0 ± 0.5	0.332

*NACT* neoadjuvant chemotherapy

Table 5 demonstrates the results for patients who received PMRT, and those who didn’t. There was no significant difference in the aesthetic outcomes of autologous reconstruction between two groups (Table 5). For implant-based reconstruction in patients receiving PMRT, the aesthetic outcomes were similar between the NACT and non-NACT groups. The only difference observed was for the symmetry of the IMF, and patients with NACT were scored significantly better than patients without NACT (3.4 ± 0.5 vs. 2.4 ± 0.2, *p* = 0.014) (Table 5). Similarly, the aesthetic outcomes were comparable between the NACT and non-NACT groups among patients without PMRT (Table 5).

**Table 5:** Aesthetic outcome among patients with and without post-mastectomy radiotherapy

**Patients with PMRT**									
	**All (*n*** **=** **32)**			**Implant-based reconstruction (*n*** **=** **9)**			**Autologous reconstruction (*n*** **=** **23)**		
**Outcome**	**NACT (*n*** **=** **21)**	**Non-NACT (*n*** **=** **11)**	***P *value**	**NACT (*n*** **=** **6)**	**Non-NACT (*n*** **=** **3)**	***P *value**	**NACT (*n*** **=** **15)**	**Non-NACT (*n*** **=** **8)**	***P *value**
Breast shape of reconstructive site	3.7 ± 0.6	3.4 ± 0.9	0.328	3.2 ± 0.5	2.6 ± 1.0	0.251	3.8 ± 0.6	3.7 ± 0.6	0.601
Symmetry of infra-mammary fold	4.0 ± 0.6	3.6 ± 0.9	0.221	3.4 ± 0.5	2.4 ± 0.2	0.014*	4.2 ± 0.5	4.0 ± 0.6	0.483
Symmetry of breast volume	3.8 ± 0.5	3.4 ± 0.7	0.058	3.5 ± 0.7	2.6 ± 0.7	0.092	3.9 ± 0.5	3.6 ± 0.4	0.214
Symmetry of breast shape	3.5 ± 0.7	3.2 ± 0.8	0.180	3.1 ± 0.8	2.3 ± 0.7	0.177	3.7 ± 0.6	3.5 ± 0.6	0.426
Overall outcomes	3.6 ± 0.7	3.4 ± 0.7	0.540	3.1 ± 0.6	2.6 ± 0.8	0.381	3.8 ± 0.6	3.8 ± 0.4	0.772
**Patients without PMRT**									
	**All (*n*** **=** **155)**			**Implant-based reconstruction (*n*** **=** **77)**			**Autologous reconstruction (*n*** **=** **78)**		
**Outcome**	**NACT (*n*** **=** **14)**	**Non-NACT (*n*** **=** **141)**	***P *value**	**NACT (*n*** **=** **9)**	**Non-NACT (*n*** **=** **68)**	***P *value**	**NACT (*n*** **=** **5)**	**Non-NACT (*n*** **=** **73)**	***P *value**
Breast shape of reconstructive site	3.5 ± 1.0	3.8 ± 0.7	0.129	3.1 ± 1.0	3.6 ± 0.8	0.089	4.3 ± 0.3	4.1 ± 0.5	0.323
Symmetry of infra-mammary fold	3.7 ± 0.9	4.0 ± 0.7	0.182	3.4 ± 0.9	3.8 ± 0.8	0.123	4.3 ± 0.4	4.2 ± 0.6	0.514
Symmetry of breast volume	3.7 ± 0.8	3.8 ± 0.7	0.380	3.4 ± 0.9	3.8 ± 0.8	0.160	4.2 ± 0.5	3.9 ± 0.6	0.273
Symmetry of breast shape	3.3 ± 0.9	3.7 ± 0.7	0.080	2.9 ± 0.9	3.5 ± 0.8	0.060	4.0 ± 0.5	3.9 ± 0.6	0.652
Overall outcomes	3.5 ± 1.0	3.8 ± 0.7	0.071	3.1 ± 1.0	3.6 ± 0.7	0.083	4.1 ± 0.6	4.1 ± 0.5	0.866

*PMRT* postmastectomy radiotherapy, *NACT* neoadjuvant chemotherapy

## Discussion

To the best of our knowledge, this is the first study that solely analyzed both aesthetic outcomes and surgical outcomes in patients who received mastectomy and immediate breast reconstruction after NACT. Although the NACT group presented with more advanced cancers and required more ALND and PMRT, the type of mastectomy (SSM versus NSM) performed was similar between the two groups, and the local recurrence rate was comparable within the limited follow-up time. Our results revealed that NACT did not increase the rate of short-term or late complications and that it was safe to perform immediate breast reconstruction after NACT, with either autologous or implant-based breast reconstruction. According to the results of the aesthetic outcome analysis, autologous reconstruction was more likely to have better aesthetic outcomes than implants, irrespective of the use of NACT.

Tables 1 and 2 revealed that various differences existed between patients with and without NACT. Patients in the NACT group was significantly younger. This could possibly originate from two reasons. First, the biological types of young-onset breast cancer are more likely to be HER-2-positive or TNBC breast cancers, which are two main indications for NACT. In addition, more multicentric breast cancers are present in young patients, which precludes breast conservation surgeries [21]. Our results indicated more HER2-positive cancer and TNBC in the NACT group, which echoed the features of young-onset breast cancer. Second, both NACT and immediate breast reconstruction were possibly more accepted among younger patients.

Patients in the NACT group presented with a more advanced cancer stage with larger tumors and/or axillary nodal involvement, even after NACT. PMRT was more frequently administered in the NACT group, which corresponded to the difference that more advanced breast cancers were in the NACT group. In addition, regarding implant-based reconstruction, the NACT group was significantly more likely to undergo two-stage reconstruction. This could be attributed to the higher requirement for PMRT because of the more advanced cancer status. To sum up, disease severity is higher in the NACT group than non-NACT group. It is reasonable that a higher rate of distant metastasis was observed in the NACT group than in the non-NACT group (19.6% versus 3.1%, *p* < 0.01). Both NACT group and non-NACT group had extremely low local recurrence rate and there was no significant difference between these two groups. The result suggested that both skin-sparing mastectomy and nipple-sparing mastectomy have good local control with the limited follow-up time.

A tendency towards more NSM than SSM was performed in the NACT group (41.3%) in comparison to the non-NACT group (30.9%), but the difference was not statistically significant (*p* = 0.226). The possible reasons might be that advances in procedures and technology, nipple-sparing mastectomy has proved to safely performed and become more popular nowadays. Besides, application of NACT is expanded and NACT is now widely provide for patients with TNBC, HER-2 positive breast cancer, and early-stage breast cancer. For those with good response to NACT, the size of the tumor greatly reduced, and more tissue preservation of the skin envelope and nipple areolar complex could be done. Thus, nipple-sparing mastectomy could possibly be more frequently performed in these NACT patients. However, there is also a tendency of favoring nipple-sparing mastectomy over skin-sparing mastectomy since the oncological safety is confirmed in patients who are eligible to receive the surgery. Therefore, the tendency of more nipple-sparing mastectomies was done in the NACT group could not be directly translated into the advantages of the delivery of NACT. Although the spare of nipple-areolar complex in NSM might potentially increase the overall complication rate considering the inclusion of complications from the preserved nipple-areolar complex, the preservation of nipple areolar complex also enhances aesthetic results along with immediate reconstruction. On the other hand, the application of NACT to enhance tissue preserving also possibly contributes to a better aesthetic outcome after surgery.

### Surgical complications

Chemotherapeutic agents target rapidly dividing cells; thus, wound healing is just as susceptible to these effects as cancer cells [22]. Considering the negative impact, surgical intervention can only be delivered a certain time after NACT. On the other hand, a longer time interval between the last course of NACT to surgery raised the issue of oncological safety. Thus, the optimal timing for surgical intervention after NACT has long been studied and debated. A recently published study by Sanford et al. in 2016 reported that patients with neoadjuvant chemotherapy to surgery intervals of up to 8 weeks had equivalent overall survival (OS), recurrence-free survival (RFS), and locoregional recurrence-free survival (LRFS) [23]. In addition, a retrospective review by Sutton et al. in 2020 concluded that the interval less than 28 days is a risk factor for postoperative wound complications [24]. Our average interval of 4.8 weeks sits in a safe time point for surgical intervention to balance the oncological safety and reduced wound healing related complications. Besides, factors impairing wound healing included comorbidities (diabetes, obesity, protein energy malnutrition), medications (steroids, NSAIDs, anti-rejection medications), oncological interventions (radiation, chemotherapy), and lifestyle habits (smoking, alcohol abuse) [11]. Studies that have investigated the complication rate of patients who received NACT with immediate breast reconstruction reported overall complication rates between 16 and 31% [17, 25–27]. Our overall complication rate between 17.5 and 21.7% was similar to that reported in most of the literature. Many previous studies indicated that the surgical complication rates are similar regardless of the use of NACT, and our results echoed these reports [14–16, 18, 25, 28–32]. Bowen et al. presented a large, matched cohort study controlled for preoperative risk factors and surgical procedures performed and concluded that breast cancer patients who received NACT had no increased risk for surgical morbidity [30]. Similarly, Beugels et al. reported comparable postoperative complication rates for patients treated with and without NACT after immediate breast reconstruction using free DIEP flap [31]. The study also adjusted for potential confounding variables, and no significant difference was identified in the multivariable models. Unlike Beugels and colleagues’ study focusing on DIEP flap breast reconstructions, our result expanded the scope of looking at both implant-based and autologous breast reconstruction, supporting the ideas of immediate breast reconstruction with different reconstruction methods. Although many studies have confirmed the equivalent safety of surgery with or without NACT, studies including evaluations of well-controlled confounding effects are limited [18, 30, 32]. Moreover, our results indicated that NACT was not a specific factor that specifically increased the incidence of complications.

Unlike most of the encouraging results, some studies reported that NACT was associated with a higher rate of surgical complications [33–35]. Mehrara et al. reviewed 952 patients undergoing immediate autologous breast reconstruction. NACT was associated with an increased rate of overall complications. Most of the associated complications of NACT, however, were minor ones, such as donor-site wound healing problems and fat necrosis in multivariate analysis [33]. In addition, recent neoadjuvant treatment often includes targeted therapy, and the negative effect of the medications could be reduced. Frey et al. reported that neoadjuvant (with or without adjuvant) chemotherapy significantly increased the risk of complications due to wound healing problems compared with that in patients treated without chemotherapy, but the analysis did not adjust for confounding effects [35]. Moreover, the study focused only on the outcomes of NSM, which is a procedure known to have slightly higher complication rates than skin-sparing mastectomy.

### Aesthetic outcome

Our results indicated comparable overall aesthetic results between patients with and without NACT for autologous tissue reconstruction. The scores of the aesthetic outcomes were generally lower for implant-based reconstructions with significant differences in overall outcome observed. In addition, autologous reconstruction was more likely to have better aesthetic outcomes than implant-based reconstruction, irrespective of the use of NACT.

Autologous breast reconstruction has several known advantages including the symmetry of breast shape, and extra skin available to fully replace the defect of the skin envelope when sacrificed, and more tolerable to radiotherapy than breast implants although radiotherapy-related complication may still happen [36]. On the other hand, complications like capsular contracture and implant rupture or exposure, may occur after radiotherapy in patients who underwent implant-based reconstruction. Our results, both in the NACT and non-NACT groups, echoed previous publications in that, breast reconstruction with autologous tissue had better aesthetic outcomes than breast implants, with minimal morbidities [37–39]. Our results further support the role of autologous breast reconstruction by providing objective satisfaction scores rated by plastic surgeons.

NACT group obtained lower scores from plastic surgeons regarding the overall aesthetic result of the implant-based reconstruction. As indicated in Table 5, the aesthetic outcomes of autologous reconstruction did not present any differences, either in patients with or without PMRT in different groups. The overall results of implant-based reconstruction presented similarly between the two groups. However, a trend towards lower satisfactory scores was observed for each item in the non-NACT group than in the NACT group following PMRT; among these items, only the difference in the symmetry of the IMF was significant. Compromised aesthetic results and complications regarding radiation and implant-based reconstruction have been addressed, including capsular contracture, infection, skin necrosis, scarring, and fibrosis [40–43]. The favorable results of implant-based reconstruction in the NACT group could be due to the fact that in patients with a good response to NACT, the size of the tumor was greatly reduced, and there was a tendency (but not a statistically significant difference) towards more tissue preservation of the skin envelope and nipple-areolar complex; thus, less tension was encountered during direct-to-implant reconstruction. Of note, the case number remained low in the group with implant-based reconstruction and PMRT. Bias may be present, and further studies with larger patient numbers should be conducted later to confirm the findings.

Jagsi et al. reported that autologous reconstruction appeared to yield better patient-reported satisfaction and a lower risk of complications than implant-based reconstruction among patients receiving PMRT [43]. Our results echoed these findings, both in NACT and non-NACT groups. Autologous breast reconstruction should be suggested to patients if PMRT is anticipated. However, implant-based reconstruction has advantages, including shortened surgical time and postoperative recovery period without donor site morbidities. If the patient did not receive radiotherapy or had a sufficient skin envelope, a good aesthetic outcome could still be obtained with implant-based breast reconstruction.

Unlike patients with PMRT, patients without PMRT in the NACT group seemed to have lower scores for the aesthetic results, but there was no significant difference. Our initial attempt to further investigate the aesthetic result of implant-based reconstruction was to identify the factors that compromised the overall aesthetic outcomes of implant-based reconstruction in the NACT group. However, our results revealed comparable overall results of implant-based reconstruction between the NACT and non-NACT groups, regardless of the presence or absence of PMRT. Taken together, we assumed that the aesthetic outcomes of implant-based reconstruction were comparable in patients with or without NACT. Of note, patients with implant-based reconstruction received either one stage direct-to-implant reconstruction or two stage reconstruction. Although one versus two stage may affect the aesthetic outcome, the case number in each group will be too small for comparison if patients were further grouped according to this. More case collection with longer follow up will be required to further confirm the results. Moreover, since some of the patients who received tissue expander insertion received autologous breast reconstruction after PMRT, this might be an associated reason that radiation associated issue was minimal in implant-based reconstruction.

Our study included analyses for both surgical complications and aesthetic outcomes, a well-controlled surgical technique, and adequate adjustment for confounding factors by statistical analysis and revealed comparable reconstruction complication rate and satisfying aesthetic outcome. NACT was a safe procedure and played a small role in the aesthetic outcomes. Although our study was the first to demonstrate a comparable complication rate and aesthetic satisfaction rate among patients receiving immediate breast reconstruction after NACT and mastectomy, several limitations exist. This was a retrospective and single-surgeon practice study with relatively small sample size. While the use of single surgeon’s cases eliminates the patient selection and surgical variables, the number of patients in each subgroup remained small, and it may not be generalizable to all practice. Moreover, patients with different indications for NACT may present different disease characteristics and preoperative staging, and the heterogeneity can possibly present. About regimens of neoadjuvant chemotherapy, variations might exist between every patient in consideration of evolution of chemotherapeutics, modifications between breast surgeons or medical oncologists who arranged chemotherapy for the patients. The 6-month to one-year follow up for aesthetic outcomes might also be insufficient even the patients who have completed PMRT without any complications and aesthetic outcome at a longer follow up time is warranted later. Furthermore, this study-specific questionnaire for aesthetic outcomes is not validated. As the safety and aesthetic results have been confirmed, a larger-scale, prospective and long-term follow-up study that includes more clinical cases can be expected. Another limitation of the study was regarding statistics that we were unable to correct the type I error rate due to the limited sample size, which may result in some false positive results. On the other hand, the achieved power is also low along with the small sample size. This again warrants future large-scaled studies to confirm the findings.

## Conclusion

Immediate breast reconstruction can be performed safely in patients with NACT. Moreover, the aesthetic outcomes of immediate breast reconstruction are comparable between patients treated with and without NACT. Convincing results support that immediate breast reconstruction can be provided safely for patients with NACT and achieve and aesthetically pleasing results.

## Supplementary Information

**Additional file 1: Appendix Table.** Questionaries of Aesthetic Outcome

## Data Availability

The datasets used and/or analyzed during the current study are available from the corresponding author on reasonable request.

## Abbreviations

NACT: Neoadjuvant chemotherapy
ALND: Axillary lymph node dissection
BCS: Breast conservation surgery
TNBC: Triple-negative breast cancer
HER-2: Human epidermal growth factor receptor-2
SSM: Skin-sparing mastectomy
NSM: Nipple-sparing mastectomy
BMI: Body mass index
pCR: Pathological complete response
SLN: Sentinel lymph node
IMF: Infra-mammary fold
PMRT: Postmastectomy radiotherapy
ORs: Odds ratios
CIs: Confidence intervals
IDC: Invasive ductal carcinoma
DCIS: Ductal carcinoma in situ
DTI: Direct-to-implant
