# Supplementary material for: Reconstructive outcome analysis of the impact of neoadjuvant chemotherapy on immediate breast reconstruction: a retrospective cross-sectional study
Source: BMC Cancer. 2021 May 8;21:522. doi: 10.1186/s12885-021-08256-y (PMC8106228; doi:10.1186/s12885-021-08256-y)
Supplement: Supplementary file 1 — Additional file 1: Appendix Table. Questionaries of Aesthetic Outcome [file 12885_2021_8256_MOESM1_ESM.docx]

**Appendix Table: Questionaries of Aesthetic Outcome**

|  | **Scores** | | | | |
| --- | --- | --- | --- | --- | --- |
| **Outcome Items** | **1 (very dissatisfied)** | **2 (dissatisfied)** | **3 (neutral)** | **4 (satisfied)** | **5 (very satisfied)** |
| **Breast shape of reconstructive site** |  |  |  |  |  |
| **Symmetry of infra-mammary fold** |  |  |  |  |  |
| **Symmetry of breast volume** |  |  |  |  |  |
| **Symmetry of breast shape** |  |  |  |  |  |
| **Overall outcomes** |  |  |  |  |  |
